# Intraoperative robotic surgical system-related problems in robot-assisted thoracoscopic surgery

**DOI:** 10.1007/s11748-024-02013-1

**Published:** 2024-03-04

**Authors:** Akira Ogihara, Motoka Omata, Hiroaki Shidei, Shota Mitsuboshi, Hiroe Aoshima, Tamami Isaka, Takako Matsumoto, Masato Kanzaki

**Affiliations:** 1 Department of Thoracic Surgery, Tokyo Women’s Medical University, 8-1 Kawada-Cho, Shinjuku-Ku, Tokyo, 162-8666 Japan; 2 Center for Medical and Nursing Education, Tokyo Women’s Medical University, 8-1 Kawada-Cho, Shinjuku-Ku, Tokyo, 162-8666 Japan

**Keywords:** Robot-assisted thoracoscopic surgery, Intraoperative trouble, Robotic surgical instrument, Robotic stapler

## Abstract

**Background:**

Malfunctions of robotic instruments during robotic surgery are well known to occur; however, detailed reports on the inherent problems associated with robotic instruments and robotic surgical systems are scarce. The objective of this study was to retrospectively investigate the intraoperative problems associated with robotic surgical systems and robotic instruments.

**Materials and methods:**

This was a single-center retrospective study. Between April 2012 and December 2022, 544 patients with consecutive lung malignancies and/or mediastinal tumors underwent robot-assisted thoracoscopic surgery. Among these, 15 cases had intraoperative problems associated with the robotic surgical system. Human error was defined as a problem caused by the incorrect operation of the robotic surgical system and human factors as problems in which the robotic surgical system stopped owing to damage to the instruments of the robotic surgical system or the self-diagnosis of the robotic surgical system. We retrospectively investigated the causes of intraoperative problems in these cases.

**Results:**

There were 4 cases (0.7%) with problems related to the robotic surgical system, 2 of which were human errors, and 11 (2.0%) with problems related to robotic surgical instruments, 6 of these were related to instruments and 5 were related to robotic staplers. Five of these were related to human factors.

**Conclusion:**

Teams performing robot-assisted thoracoscopic surgery should be familiar with the features of robotic surgical systems and various robotic devices, be aware of reported problems during robot-assisted thoracoscopic surgery, and be prepared for emergencies.

## Introduction

In recent years, robotic surgery, which has revolutionized surgery, has emerged all over the world [1]. Especially in Japan, the application of robot-assisted thoracoscopic surgery (RATS) for malignant lung tumors and mediastinal tumors by the National Health Insurance started in 2018, and the rise of robotic surgery has been remarkable [2, 3]. A main advantage of robotic surgery is its distinct precision. On the other hand, with increasing robotic surgical cases, inherent robotic surgical system (RS)-related problems have been encountered. Malfunctions of robotic instruments during robotic surgery are well known, although detailed reports on the inherent problems associated with robotic instruments and RSs are scarce. The objective of this study was to retrospectively investigate the intraoperative problems associated with robotic surgical systems (RSs) and robotic instruments.

## Materials and methods

This single-center, retrospective study was conducted at Tokyo Women’s Medical University Hospital, Department of Thoracic Surgery. The medical data of the patients were obtained from the hospital database. Written informed consent was obtained from each patient. The study protocol was approved by the Human Ethics Committee of Tokyo Women's Medical University (No. 5637). The study was conducted in accordance with the principles of the Declaration of Helsinki. Between April 2012 and December 2022, 544 patients with consecutive lung malignancies and/or mediastinal tumors underwent RATS. Among these, there were 15 cases with intraoperative problems associated with RS.

We retrospectively investigated the causes of intraoperative problems in these patients. Intraoperative problems were classified into two categories: (1) system-related problems; and (2) robot instrument-related problems. Moreover, system-related problems are defined as sudden system stoppage and system malfunction, and robot instrument-related problems are defined as instruments and staples. The total number of RATS procedures performed in our department was 21 between 2012 and 2017, 69 in 2018, 110 in 2019, 104 in 2020, 121 in 2021, and 109 in 2022.

**Fig. 1:**
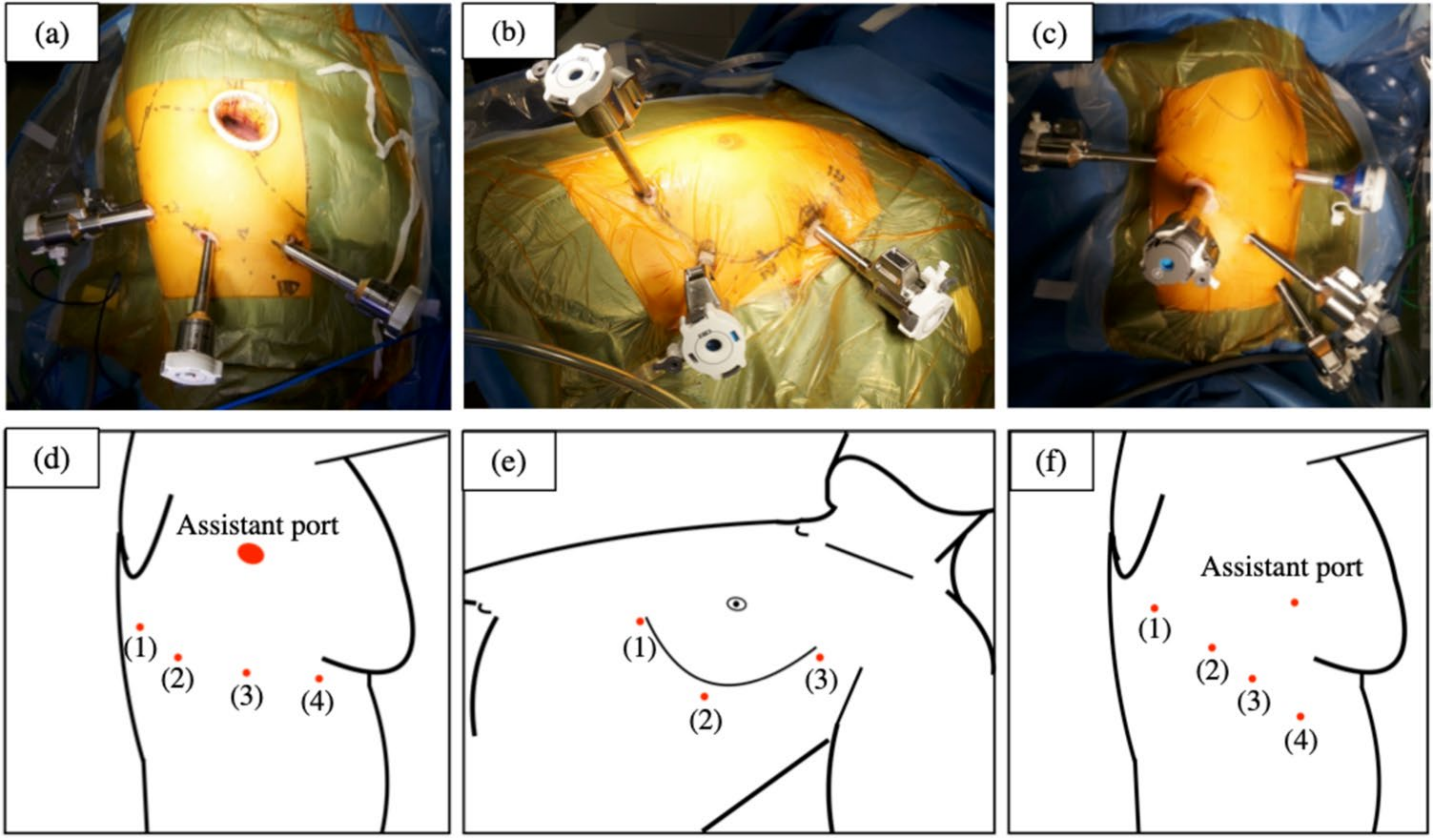
Skin incision location and port placement. **a** A photograph captured during robot-assisted thoracoscopic right lobectomy. **b** A photograph captured during robot-assisted anterior mediastinal tumor resection. **c** A photograph captured during robot-assisted thoracoscopic middle and posterior mediastinal tumor resection. **d** The schematic illustration shows that the assistant port is placed in the 4th intercostal space for upper and middle lobectomy and in the 5th intercostal space for lower lobectomy. Robotic instrument ports (1) and (2) are placed in the 9th intercostal space, port (3) in the 8th intercostal space, and port (4) is placed in the 7th intercostal space. Ports (1) and (3) are 8-mm ports, while ports (2) and (4) are 12-mm ports. An 8-mm 30° robotic camera is inserted into the thoracic cavity through port (3). **e** The schematic illustration shows port placement for anterior mediastinal tumor resection. Generally, robotic instrument ports (1) and (2) are placed in the 5th or 6th intercostal space. Port (3) is placed in the third intercostal space, and an 8-mm 30° robotic camera is inserted into the thoracic cavity through port (3). **f** The schematic illustration shows port placement for middle and posterior mediastinal tumor resection. An assistant port is placed in the 4th intercostal space and used as a port for insufflation of carbon dioxide. All four ports are placed in the 8th intercostal space. An 8-mm 30° robotic camera is inserted into the thoracic cavity through port (3)

Our surgical procedures for RATS were previously described [2, 4–7]. RATS was performed using the daVinci® Robotic Surgery System (Intuitive Surgery, Sunnyvale, CA, USA). Prior to March 2018, only the da Vinci Surgical System Type S (Intuitive Surgical, Sunnyvale, CA, USA) was used. In March 2018, either Si (Intuitive Surgical, Sunnyvale, CA, USA) or Xi (Intuitive Surgical, Sunnyvale, CA, USA) was used. From April 2018, only Xi was used. We performed RATS lobectomy with four port incisions and a 3-cm utility thoracotomy or CO_2_ insufflation combined with an assistant port. On the other hand, mediastinal tumor (MT) resection is performed with a 3-port incision for anterior MT and a 4-port incision for middle and posterior MT under CO_2_ insufflation (Fig. 1).

### Statistical analyses

Continuous data are presented as the median and interquartile range (IQR), and categorical data are presented as numbers and percentages. ﻿

## Results

The patients’ characteristics are shown in Table 1. The numbers of cases problems each year were as follows: 2018 (*n* = 2), 2019 (*n* = 4), 2020 (*n* = 1), 2021 (*n* = 4), and 2022 (*n* = 4). All 15 problems occurred with the Type Xi system.

**Table 1:** Patients' characteristics in cases with intraoperative problems

Variable	*n* (%)
Age, median (years)	66 (range36-78)
Gender	
Male	5 (33.3)
Female	10 (66.7)
Diagnosis	
Lung cancer	
Ad	7 (46.7)
Sq	1 (6.7)
SCLC	1 (6.7)
MLT	1 (6.7)
MT	
Thymoma	4 (26.7)
Thymic cyst	1 (6.7)

Ad, adenocarcinoma; Sq, squamous cell carcinoma; SCLC, small cell lung carcinoma; MLT, Metastatic lung tumor; MT, mediastinal tumor

### Intraoperative problems

#### 1. System-related problems

There were four problems related to RS.

(i) System suddenly stoppage

Of the two sudden stoppages of the system, one was due to the RS detecting abnormal energization when the patient-side doctor used an electric scalpel; in this case, the RS stopped immediately, and the other was due to the system power cable being unplugged (Table 2).

**Table 2:** Perioperative outcomes in cases with intraoperative problems

Variable	*n* (%)
Surgical procedure	15 (100)
Lobectomy	8 (53.5)
Segmentectomy	2 (13.3)
Thymo-thymectomy	2 (13.3)
MT resection	3 (20.0)
Complication	
Intraoperative	0
Postoperative	5 (33.3)
Atelectasis	1 (6.7)
Bronchial asthma	1 (6.7)
NSVT	1 (6.7)
SE	1 (6.7)
Cerebral infarction	1 (6.7)

*MT* mediastinal tumor, *BA* bronchial asthma, *NSVT* Non-sustained ventricular tachycardia, *SE* subcutaneous emphysema

(ii) System malfunction

In two cases, soon after the RATS console was started, the console surgeon noticed that the robotic instrument was not moving smoothly. After checking for damage to the robotic instrument and the depth of the port, the problem could not be identified; therefore, the system was restarted, and the robot instrument’s movement became normal. However, the cause of this phenomenon remains unknown.

These four problems were resolved by restarting the RS, and we were able to resume operations.

#### Robot instrument-related problems

There were 11 cases of problems related to robotic surgical instruments, 6 of these were related to instruments and 5 were related to robotic staplers. Five of these factors were also shown to be related to human factors.

(i) Robotic instrument failure

**Fig. 2:**
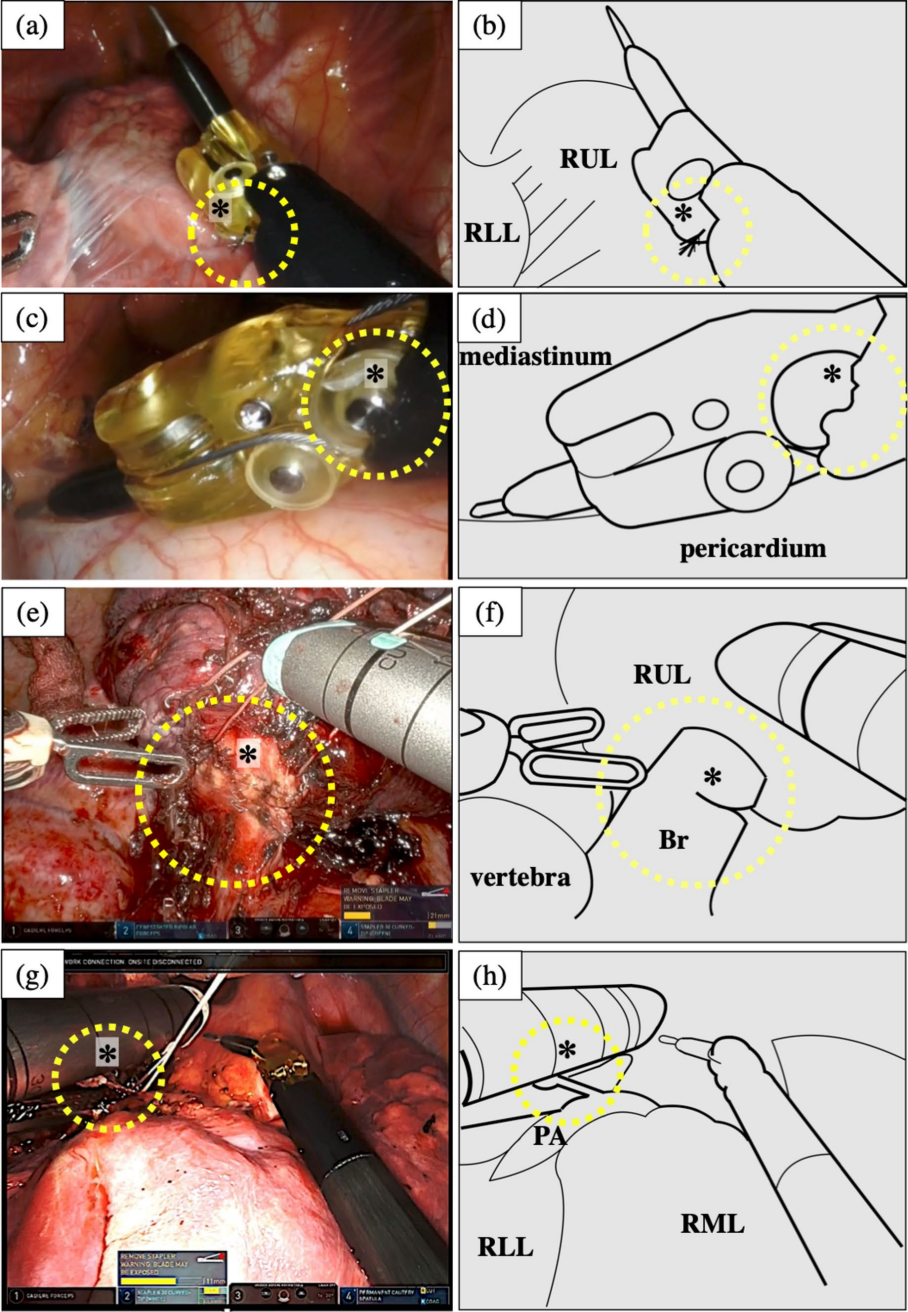
Intraoperative photographs of problems related to robotic instruments. *RUL* right upper lobe, *RLL* right lower lobe, *Br* bronchus, *PA* pulmonary artery, *RML* right middle lobe; All photographs were extracted from digital video data taken during RATS. **a** A photograph showing the disconnection of the wire of the permanent cautery spatula (yellow dotted circle and asterisk). During RATS mediastinal tumor resection, the console surgeon noticed that the joint range of motion of the spatula was restricted during the removal of adhesions, and a problem was discovered. **b** Schematic illustration. The disconnection of the wire of the permanent cautery spatula (yellow dotted circle and asterisk). **c** A photograph showing the plastic breakage at the tip of the permanent cautery spatula (yellow dotted circle and asterisk). During RATS mediastinal tumor resection, the patient-side doctor noticed that breakage, and the spatula needed to be replaced. **d** A schematic illustration indicates the area of breakage (yellow dotted circle and asterisk). **e** A photograph showing failed transection of the upper lobe bronchus (yellow dotted circle and asterisk). During RATS right upper lobe resection, the blade stopped while dissecting the upper lobe bronchus due to stapler teeth that had fallen into the thoracic cavity in the groove in which the blade was running were bitten, causing the blade to stop. **f** A schematic illustration showing that the bronchial stump could not be transected due to stapler trouble (yellow dotted circle and asterisk). **g** A photograph showing a misfire during stapler pulmonary artery resection (yellow dotted circle and asterisk). During RATS right upper lobe resection, the blade stopped while performing stapling of the superior arterial trunk and the dissection was stopped. **h** A schematic illustration showing that the arterial stump could not be dissected due to stapler trouble (yellow dotted circle and asterisk)

Disconnection of the wire was observed in 3 cases, including 1 case with a permanent cautery spatula (Fig. 2a, b) and 2 cases with fenestrated bipolar forceps. There was one case each of plastic breakage at the tip of the permanent cautery spatula, (Fig. 2c, d), disconnection of the bipolar cord, and deformation that prevented the tip of the fenestrated bipolar forceps from being gripped normally. In all cases, it was necessary to use alternative instruments.

(ii) Misfire of robotic stapler

Of the five cases of robotic stapler-related problems, three cases involved failure to transect the bronchi, 2 involved failure to transect the pulmonary artery, and 1 of the pulmonary artery misfires involved blade stoppage during resection of the pulmonary artery (Fig. 2d, e, f, g). These problems required additional transection with a linear mechanical stapler by the patient-side doctor. No bleeding occurred during pulmonary artery transection.

None of the 15 patients who had problems underwent emergent conversion to thoracotomy, and there was no 30-day or 90-day mortality.

## Discussion

Advances in science and technology and their development are essential in modern society. In medicine, especially in surgery, advances in surgical instruments can change the nature of surgery. In recent years, robotic surgery has taken the world by storm, revolutionizing surgery [1]. The main points that surgeons want from a surgical robot system are the ability to perform difficult surgeries easily, with highly accurate surgical techniques, with a consistent quality of surgery that does not differ depending on the surgeon, and a short operative time [2, 4].

Since 2018, RATS surgery has been covered under Japan's National Health Insurance system, and the number of surgical cases is increasing rapidly [2, 3]. The well-known “da Vinci Surgical System” (Intuitive Surgical) is an RS that has articulated robotic arms and a high-definition three-dimensional (3D) video monitoring system. The benefits of RS motion scaling, magnification, and articulated robotic instruments have been reported previously reported [2, 3, 8]. Interestingly, video games are gaining popularity among the younger generations, and such hobbies have potential applications in surgical skills education as an adjunctive training method. Indeed, a history of playing games and training with video games has been reported to be beneficial in robotic surgery [9]. In other words, the use of robotic surgery is expected to further increase, especially among young surgeons.

RS self-diagnoses motion and mechanical conditions during operation. If the RS determines that something is wrong, it notifies the console surgeon and patient-side doctors of the error. If the RS determines that there is an abnormality, a yellow LED on the patient cart blinks to notify that it can be handled without turning off the power while a red LED blinks if it must shut down, and the RS displays system malfunctions on the Surgeon Console display and notifies the console surgeon. The Vision Cart displays system malfunctions on the Vision Cart touch screen monitor for other surgeons. In this study, when transecting the pulmonary artery, the stapler sensed other staples that had fallen off and stopped working due to the self-diagnosis function.

O'Connor et al. reported that 5–15% of all hospital admissions involve some degree of error, and 45% of errors occur in the operating theater [10]. Although the paper did not specifically mention errors related to surgical systems and their instruments, it is clear that there are many potential opportunities for errors to occur in any patient on a daily basis. In future, we will also have to think about errors related to RS.

In the present study, we noted 2 human errors (0.4%) and 5 human factors (0.9%), which are very low numbers. The human errors included two problems related to the system, and the human factors included one problem due to careless operation by the console surgeon who broke the plastic part of the monopolar cautery instrument when removing deposits on the right-side monopolar cautery instrument with the left-side bipolar forceps; one involving thickened bronchi that could not be clamped with a stapler; and three involving stopping cutting of the pulmonary artery and bronchus cutting because of the self-diagnosis function of the system. Staplers were used to cut other tissues by replacing the cartridges.

Notably, the stoppage of cutting due to the system's self-diagnostic function was part of the normal operation of the RS, and it was classified as a human factor rather than a problem with the RS, as it involved the incorrect selection of tooth thickness relative to tissue thickness, resulting in stapler teeth that had fallen into the thoracic cavity in the groove in which the blade was running being bitten and thereby causing the blade to stop. For problems involving robotic instruments, although the ability to repeatedly use these instruments is an advantage, it is possible that surgical forceps may break due to wear and deterioration. However, disconnection was observed even with forceps that were used infrequently, suggesting that the operation might have exceeded the limits of the range of motion of the forceps, extraction might have been performed without straightening the tips of the forceps, or interference might have occurred between forceps. In larger hospitals, multiple surgeons perform various robotic surgeries. Therefore, it is expected that robot instruments that are used repeatedly will be damaged, causing issues such as robotic instrument deformation.

For safe RATS, it is important to share information among all members of the team, including the surgeon, assistants, nurses, and clinical engineers during surgery. However, the same error was not observed. As experience with the da Vinci system has increased, the human factors have decreased. If the members are familiar with the functions of the RS and various robotic devices, it will be possible to avoid the many of the problems that were reported during RATS procedures by preparing for emergencies.

This study was associated with some limitations. This study has a single-center, retrospective design with a small sample size. In particular, the number of patients who received RATS is still small, and there was a patient selection bias. Therefore, the results need to be confirmed in further, multicenter, large-scale prospective, and randomized studies.

## Conclusion

RATS is performed by a team of console surgeons, bedside doctors, nurses, and clinical engineers. In RATS, it is important to share information, such as the situation during surgery, with all members of the team. All members must be familiar with the features of the RS and various robotic devices as well as the problems reported here in order to be prepared for emergencies.

The abstract of this study received the Best Poster Award at the 75th annual scientific meeting of the Japanese Association for Thoracic Surgery.

## Data availability

Not applicable.
